# Surgical Treatment of Severe Neglected Congenital Muscular Torticollis in an 8‐Year‐Old Child: A Case Report

**DOI:** 10.1155/cro/5642692

**Published:** 2026-05-20

**Authors:** Bujar Shabani, Dafina Bytyqi, Leotrim Berisha, Cen Bytyqi

**Affiliations:** ^1^ Department of Orthopedics, Department of Pathophysiology, Faculty of Medicine, University of Prishtina “Hasan Prishtina”, Prishtina, Kosovo, uni-pr.edu; ^2^ Clinic of Orthopedics and Traumatology, University Clinical Center of Kosova, Prishtina, Kosovo, shskuk.org

**Keywords:** bipolar tenotomy, congenital muscular torticollis, neglected torticollis

## Abstract

**Introduction and Importance:**

Congenital muscular torticollis (CMT) represents the third most common musculoskeletal deformity encountered in pediatric practice, characterized by unilateral shortening of the sternocleidomastoid (SCM) muscle. Neglected, untreated cases are rare. The objective was to assess the surgical outcome following complete bipolar tenotomy for the management of severe, neglected left‐sided CMT in an 8‐year‐old girl.

**Case Presentation:**

An 8‐year‐old girl presented with marked facial asymmetry with left hemifacial hypoplasia, restricted cervical range of motion, and a tense, contracted left SCM. The patient underwent a complete bipolar tenotomy of the SCM. Intraoperatively, correction of the cervicomandibular angle (CMA) was achieved, with a reduction from 38° preoperatively to 8.9° postoperatively. The postoperative protocol included the use of a cervical collar and 6 weeks of intensive physiotherapy. Five years postoperatively, she maintained a neutral head position with full cervical motion and no recurrence.

**Clinical Discussion:**

Around 10%–20% of cases with CMT remain unresponsive to conservative approaches and require surgical intervention. Because of concerns regarding residual deformity and scarring, surgical correction of CMT is generally recommended between 1 and 5 years of age. However, evidence suggests that even patients treated later, like in our case at age eight, experience significant improvement in appearance, function, and pain.

**Conclusion:**

Complete bipolar tenotomy represents a safe and effective surgical technique for the management of severe, neglected CMT, leading to significant functional and cosmetic improvement even beyond the traditionally recommended age for intervention.

## 1. Introduction

Congenital muscular torticollis (CMT) is characterized by unilateral fibrotic infiltration of the sternocleidomastoid muscle (SCM), manifesting as muscular hypertrophy and contracture with subsequent limitation of cervical mobility [1–3].

When therapeutic intervention is delayed or inadequate, CMT follows a progressive clinical course with long‐term sequelae as progressive craniofacial asymmetry with alterations in hemifacial dimensions and mandibular growth trajectories and compensatory spinal curvature abnormalities that demonstrate age‐dependent progression rates [4, 5].

The current treatment paradigm advocates initial conservative management with manual stretching protocols and therapeutic positioning. Surgical intervention becomes indicated when deformity persists beyond 12 months of age despite compliant nonoperative management. There is substantial consensus within the literature that optimal surgical outcomes correlate with earlier intervention, with significantly superior functional and cosmetic results achieved when surgical release is performed between 12–48 months of age [6, 7]. However, in low‐resource or war and conflict settings or due to delays in diagnosis, patients may present with neglected CMT in later childhood.

The purpose of this study was to present a rare case with severe neglected CMT treated with bipolar tenotomy and report the clinical results at 5‐year follow‐up.

This case report has been reported in line with the CARE checklist [8].

The educational value of this case report consists of two main points: first, the demonstration of the consequences of the natural history of untreated CMT and second, even when the surgery is done beyond the recommended age the results can be good.

## 2. Presentation of Case

### 2.1. Case Description

**Figure 1:**
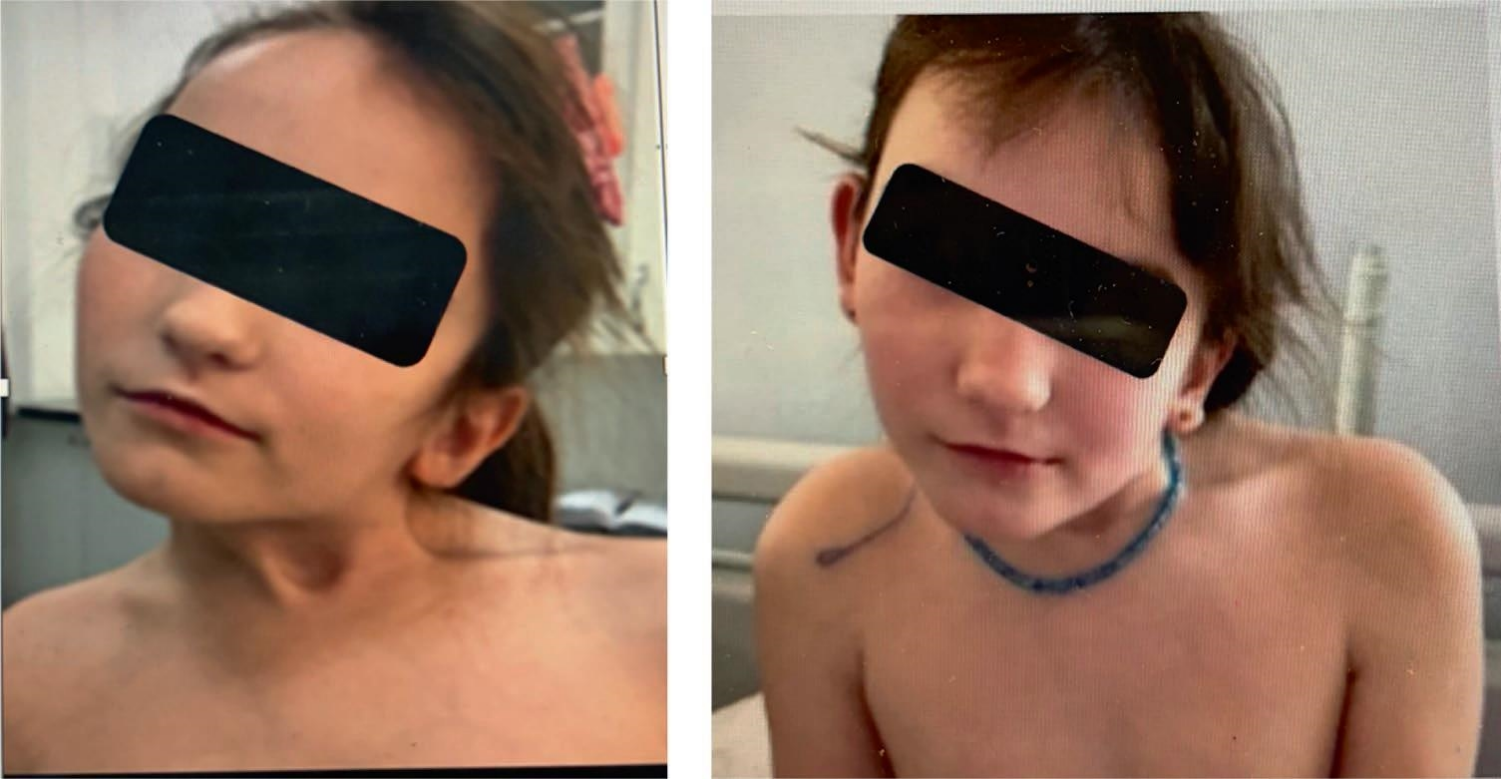
First time presentation: left‐sided head tilt, contralateral (right‐sided) rotation of the chin, and cervical stiffness.

An 8‐year‐old girl was presented to the Pediatric Orthopedic Reconstructive Department with progressive left‐sided head tilt, contralateral (right‐sided) rotation of the chin, and cervical stiffness. The patient′s primary concerns were functional limitations, cosmetic deformity, and cervical spine discomfort (Figure 1).

**Figure 2:**
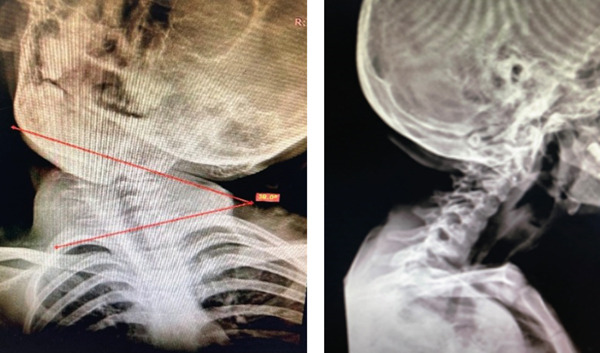
The preoperative anteroposterior and lateral plain radiographs show a 38^0^ cervicomandibular angle of cervical part of spine.

Physical examination revealed a tense and contracted left SCM, with significant deficits in both active and passive cervical rotation (55^0^) and lateral flexion (60^0^). Craniofacial asymmetry was notable, with marked flattening of the right occipital region. Radiographic evaluation demonstrated a cervicomandibular angle (CMA) of 38^0^ (Figure 2). Cervical vertebral anomalies were excluded. The patient consulted the ophthalmologist, ear–throat–neck specialist, and *neurosurgeon* to rule out nonmuscular and *intracranial* causes of torticollis.

The score in Cheng and Tang′s scoring system for assessment of clinical and subjective outcome in CMT was poor, 3 points [4].

Despite an intensive course of passive and active stretching exercises, outcomes were unsatisfactory due to the severity of the deformity.

#### 2.1.1. Family History

She is the third of 5five children; none of the other family members had relevant anomalies. The child was born during a period of war in her country, which may have contributed to the delay in seeking treatment.

Given the functional and cosmetic impairment and failure of conservative management, surgical intervention was indicated. The patient was evaluated preoperatively and postoperatively using the Cheng and Tang scoring system [8].

### 2.2. Surgical Procedure

The procedure was performed by an experienced pediatric orthopedic surgeon. Under general anesthesia, the patient was positioned supine with a towel under the thorax and the neck gently extended and rotated contralaterally to accentuate SCM tightness. Because of severe contracture, a complete bipolar tenotomy was performed.

#### 2.2.1. Proximal Release

**Figure 3:**
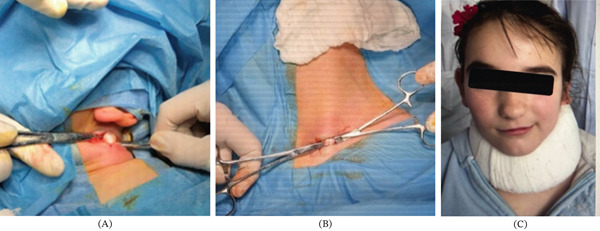
(A) Proximal release, (B) distal release, and (C) the patient wore a cervical collar with an elevated platform on the operated side for 6 weeks.

A 2–2.5‐cm longitudinal incision was made distal to the mastoid tip to release the proximal SCM attachment while safeguarding the facial and accessory nerves (Figure 3A).

#### 2.2.2. Distal Release

A second 2–3 cm incision was made over the medial clavicle to release the sternal and clavicular heads of the SCM (Figure 3B).

Care was taken to avoid injury to the carotid sheath and major neurovascular structures. The wounds were closed with 4–0 Vicryl Rapide sutures.

No intraoperative or postoperative complications, such as nerve injury or wound infection, were observed. The patient was discharged on postoperative Day 2. Postoperatively, the patient wore a cervical collar with an elevated platform on the operated side for 6 weeks (Figure 3C). Early gentle range‐of‐motion exercises were initiated once surgical pain subsided. The patient had 6 weeks of physical therapy (3 times a week) and then 4 more weeks (at‐home exercises with supervision of a therapist once a week).

### 2.3. Outcomes

**Figure 4:**
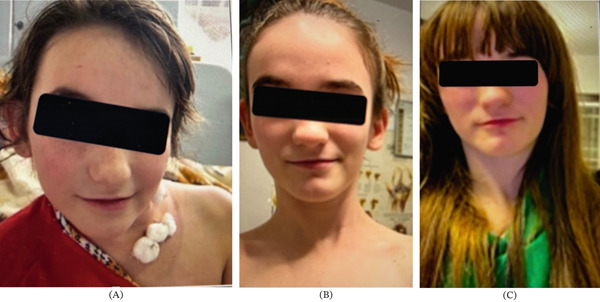
(A) Seven days–postoperatively, (B) 3 years postoperatively, and (C) 5 years postoperatively, ∗note the improvement of the face asymmetry.

Serial follow‐up showed progressive improvement. At 3 months (Figure 4A) and subsequently at 3 and 5 years postoperatively (Figure 4B,C), the patient demonstrated excellent head posture with minimal residual tilt.

**Figure 5:**
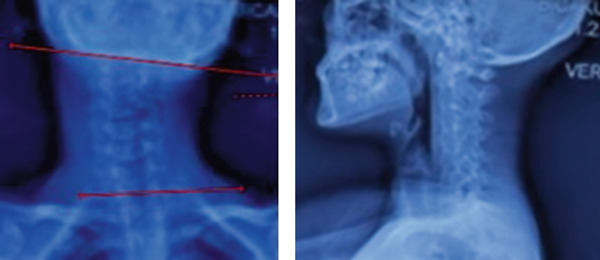
Last x‐rays show the CMA angle of 8.9^0^.

The final clinical examination showed rotational deficit of 5^0^; lateral flexion deficit was 7^0^. Radiographs demonstrated a reduction of the CMA from 38° to 8.9° (Figure 5).

The asymmetry of face and head was mild. Patient and her parents were satisfied with the final result, scoring it as excellent, commenting also on the improvement in her social and scholar aspect of life. The patient′s quality of life and functional abilities were significantly improved (Table 1) (Table 2).

**Table 1:** Cheng and Tang′s scoring presurgery and 5 years follow‐up after surgery; 16–18 p Excellent, 12–15 p Good, 6–11 p Average/Fair, < 6 points = Poor.

Results	Preoperatively	5 years follow‐up postoperatively
Rotational deficit	55 (0 points)	5 (3 points)
Lateral flexion deficit	60 (0 points)	7 (2 points)
Craniofacial asymmetry	Severe (0 points)	Mild (2 points)
Scar	None (3 points)	Mild (2 points
Residual contracture	Clavicular/sternal (0 points)	None (3 points)
Subjective assessment	Poor (0 points)	Excellent (3 points)
Head tilt	38 (0 points)	8.9 (2 points)
Overall score	Poor (3 points)	17 (Excellent)

**Table 2:** The timeline of treatment flow of the case.

Time point	Key details/documentation points
Birth/early infancy	‐ No early physiotherapy or diagnostic imaging performed.
	‐ Parents noted subtle head tilt but no intervention initiated.
School age 8 years old	‐ Presentation with neglected CMT
	‐ Physical exam findings: fibrotic SCM band, decreased ROM.
	‐ Radiographs exclude osseous pathology.
Preoperative evaluation	‐ Multidisciplinary assessment (orthopedic surgery, ophthalmology ant ENT consultation).
	‐ Cheng and Tang score documented as 3 (poor).
Surgery (Day 0)	‐ Complete bipolar SCM release/tenotomy.
Rehabilitation (0–6 weeks)	‐ Early rehabilitation initiation.
	‐ Use of soft cervical collar to maintain alignment.
Intermediate follow‐up (3 months)	‐ Functional recovery assessment.
	‐ Improved ROM and symmetry.
Final outcome (5 years post‐op)	‐ Full or near‐full neck mobility maintained.
	‐ Cheng and Tang score 17 (excellent).
	‐ High functional and cosmetic satisfaction.
	‐ No recurrence or complications noted.

## 3. Discussion

Incidence rates of CMT range from 0.3% to as high as 16%, making it one of the most common congenital conditions in the pediatric population [8, 9]. Although most cases resolve spontaneously or with conservative treatment, severe fibrosis may lead to persistent deformity.

Sönmez et al. found that 95% of patients diagnosed and treated effectively before age 1 year did not need surgical treatment [9]. Approximately 10%–20% of cases are refractory to conservative management [10], warranting surgical intervention before school age [3]. The condition is notable for its potential long‐term developmental complications, including facial asymmetry and motor delays, if left untreated. Surgical indications for CMT include significant muscle tightness, persistent facial asymmetry, and failure of conservative treatments, often necessitating timely intervention to prevent complications. It is well known that the optimal age of surgery is before age of 5, because the SCM and surrounding soft tissues have higher elasticity and a higher remodeling rate that prevents the development of secondary craniofacial and cervical skeletal asymmetries [11]. In our case, the surgery was done at the age of 8, and the result was very good. Although concerns have been raised regarding residual deformity and scarring when surgery is performed after age 5 [12], a systematic review of 220 cases showed that 81% of patients experienced improvements in appearance, pain, and function following surgery [13]. Notably, outcomes did not significantly differ between patients above and below 15 years of age [14]. Cheng et al. found that the most important factor affecting the overall result and outcome was the age of the patient at the time of operation. However, the study showed that for patients who were older than 10 years, most had excellent results indicating the benefit of surgery even in the late cases [13]. One limitation of the measured outcomes is that some of the items on the Cheng and Tang score are subjective, such as scar or contracture assessment, but they were correlated with objective measures also.

## 4. Conclusion

Our findings further support the efficacy of surgical treatment in patients with neglected CMT. Intraoperative release of SCM tension led to immediate improvements in cervical rotation and lateral flexion. The bipolar surgical approach offers several advantages over traditional unipolar techniques in the treatment of neglected torticollis. The precision of bipolar coagulation allows for controlled sectioning of hypertrophic and contracted muscles while minimizing thermal damage to surrounding tissues. This is particularly crucial in neglected cases where anatomical landmarks may be distorted due to chronic contracture and fibrosis. The ability to achieve hemostasis with minimal collateral tissue damage reduces postoperative complications and promotes faster healing.

Beyond physical improvement, our patient reported enhanced psychosocial well‐being postoperatively. This may reflect the significant emotional burden of chronic deformity, particularly in adolescent females facing cultural and social pressures.

## Author Contributions

Bujar Shabani: conceptualization and methodology. Dafina Bytyqi: writing and data curation. Leotrim Berisha: data curation. Cen Bytyqi: validation and writing review.

## Funding

No funding was received for this manuscript.

## Ethics Statement

Based on Ethical Regulation of Faculty of Medicine, ethical approval was not required for this case report as the intervention performed is an established technique and the patient/family provided written informed consent.

## Consent

The patient allowed personal data processing and informed consent was obtained from the individual participant included in the study.

## Conflicts of Interest

The authors declare no conflicts of interest.

## Supporting information


**Supporting Information** Additional supporting information can be found online in the Supporting Information section. CARE checklist.

## Data Availability

The data that support the findings of this study are available on request from the corresponding author. The data are not publicly available due to privacy or ethical restrictions.
