# Supplementary material for: Surgical Treatment of Severe Neglected Congenital Muscular Torticollis in an 8‐Year‐Old Child: A Case Report
Source: Case Rep Orthop. 2026 May 20;2026:5642692. doi: 10.1155/cro/5642692 (PMC13189499; doi:10.1155/cro/5642692)
Supplement: Supplementary file 1 — Supporting Information Additional supporting information can be found online in the Supporting Information section. CARE checklist. [file CRO-2026-5642692-s001.pdf]

CARE case report guidelines

CARE Checklist of information to include when writing a case report

(cc) BY-NC-ND

Topic	Item	Checklist item description	Reported on Line
Title	1	The diagnosis or intervention of primary focus followed by the words "case report"	yes
Key Words	2	2 to 5 key words that identify diagnoses or interventions in this case report, including "case report"	yes
Abstract (no references)	3a	Introduction: What is unique about this case and what does it add to the scientific literature?	6
	3b	Main symptoms and/or important clinical findings	10-11
	3c	The main diagnoses, therapeutic interventions, and outcomes	11-13
	3d	Conclusion—What is the main "take-away" lesson(s) from this case?	24-26
Introduction	4	One or two paragraphs summarizing why this case is unique (**may include references**)	20-21
Patient Information	5a	De-identified patient specific information.	yes
	5b	Primary concerns and symptoms of the patient.	76-79
	5c	Medical, family, and psycho-social history including relevant genetic information	84-90
	5d	Relevant past interventions with outcomes	91
Clinical Findings	6	Describe significant physical examination (PE) and important clinical findings.	84-90
Timeline	7	Historical and current information from this episode of care organized as a timeline	yes
Diagnostic Assessment	8a	Diagnostic testing (such as PE, laboratory testing, imaging, surveys).	yes
	8b	Diagnostic challenges (such as access to testing, financial, or cultural)	87-89
	8c	Diagnosis (including other diagnoses considered)	87-88
	8d	Prognosis (such as staging in oncology) where applicable	yes
Therapeutic Intervention	9a	Types of therapeutic intervention (such as pharmacologic, surgical, preventive, self-care)	105-109
	9b	Administration of therapeutic intervention (such as dosage, strength, duration)	110-121
	9c	Changes in therapeutic intervention (with rationale)	110-121
Follow-up and Outcomes	10a	Clinician and patient-assessed outcomes (if available)	127-134
	10b	Important follow-up diagnostic and other test results	134-135
	10c	Intervention adherence and tolerability (How was this assessed?)	-
	10d	Adverse and unanticipated events	122
Discussion	11a	A scientific discussion of the strengths AND limitations associated with this case report	157-179
	11b	Discussion of the relevant medical literature **with references**.	157-179
	11c	The scientific rationale for any conclusions (including assessment of possible causes)	-182-190
	11d	The primary "take-away" lessons of this case report (without references) in a one paragraph conclusion	182
Patient Perspective	12	The patient should share their perspective in one to two paragraphs on the treatment(s) they received	191-193
Informed Consent	13	Did the patient give informed consent? Please provide if requested	Yes ☑ yes No ☐
